# The Genetic Diversity of White-Backed Planthoppers (*Sogatella furcifera*) between Myanmar and Yunnan Province of China

**DOI:** 10.3390/genes14122164

**Published:** 2023-11-30

**Authors:** Yue Liu, Khin Nyein Chan, Xiangyong Li, Xueqing Zhao, Dong Chu, Yanqiong Yin, Ying Liu, Aidong Chen

**Affiliations:** 1 Key Laboratory of Green Prevention and Control of Agricultural Transboundary Pests of Yunnan Province/Agricultural Environment and Resource Research Institute, Yunnan Academy of Agricultural Sciences, Kunming 650205, Chinayinyq1977@sina.com (Y.Y.);; 2 State Key Laboratory of Biocatalysis and Enzyme Engineering, School of Life Sciences, Hubei University, Wuhan 430062, China; 3 Biotechnology Research Department, Ministry of Education, Mandalay 05151, Myanmar; 4 Key Lab of Integrated Crop Pest Management of Shandong Province, College of Plant Health and Medicine, Qingdao Agricultural University, Qingdao 266109, China

**Keywords:** mitochondrial DNA, population structure, gene flow, *Sogatella furcifera*

## Abstract

In order to clarify the migration route and the source of white-backed planthopper (WBPH) (*Sogatella furcifera*) between Myanmar and Yunnan Province, China, we collected six populations throughout Myanmar and five populations around the border areas in Yunnan Province, China. A total of 790 base pairs in the mtDNA COI genes from 416 individuals were obtained. A total of 43 haplotypes were identified, among which 37 were unique haplotypes, and the remaining 6 were shared among different populations. Two common shared haplotypes (H_1 and H_2) had a widespread distribution in all populations and accounted for 88.8% of the total haplotype frequency, suggesting a high-level gene flow among the Myanmar and Yunnan populations. Bayesian skyline plot (BSP) analysis results indicated that the effective population size of WBPH expanded between about 10,000 and 7000 years ago, and *S. furcifera* might follow the post-LGM (Last Glacial Maximum) expansion pattern. Based on the total migrant (*N_e_m*) value, it can be deduced that north and northeast Myanmar were the primary migration sources for WBPH populations in the southwest and south Yunnan regions. This study aims to contribute to the sustainable regional management of this important rice pest and provide new insights into the genetic diversity of WBPH in Southeast Asia.

## 1. Introduction

The white-backed planthopper, *Sogatella furcifera* (Horváth), belongs to Hemiptera: Delphacidae and is one of the most serious insect pests in rice production [1,2]. It is widely distributed in South, Southeast, and East Asia, as well as the South Pacific islands and Australia [3]. The white-backed planthopper (WBPH) possess formidable migratory abilities, enabling them to cover vast distances between different seasons and regions, spanning thousands of kilometers [4,5,6,7]. This migratory capability allows them to swiftly move from one area to another in a relatively short period, posing a significant threat to crops. The threat to rice primarily manifests during the process of feeding on plant sap, causing abnormal plant growth and development that leads to reduced yields or even plant death. Additionally, by transmitting plant viruses, such as the *Southern rice black-streaked dwarf virus* [7,8,9,10], the WBPH further exacerbates its harm to crops. This can result in significant economic losses, with estimated reductions in crop yield ranging from 10% to 30% [11], posing a serious threat to agricultural production [12]. Therefore, understanding the migration patterns of the WBPH is crucial for the development of effective regional management and prevention strategies.

In recent years, researchers have also made some progress in exploring the migration origins of the WBPH through trajectory analysis [13,14,15]. According to research by Chaoxing Hu et al., WBPH are unable to overwinter in temperate regions. Annually, the primary WBPH originate from warmer areas such as the Indochinese Peninsula, and their typical migration pattern usually begins around March annually [16]. This migration marks the peak of their movement from tropical regions into China. Regarding the migration of WBPH in Yunnan Province, it is speculated that from April to early May, these planthoppers primarily choose Myanmar as their migration source. However, by mid-May, it is confirmed that the main source of these migrating insects is the northern regions of Vietnam. This migration process is, to some extent, influenced by the seasons and climate, forming an organized and sustained migration chain [15,17]. Despite some evidence based on molecular marker analysis [18,19], our understanding of the genetic flow and migration patterns of the WBPH between Myanmar and Yunnan Province, China remains limited.

In our previous research, we found that the main migration sources of the WBPH in Yunnan Province, China include Myanmar, Vietnam, Laos, and Cambodia [13,20,21,22]. However, it is important to note that our previous studies in Myanmar primarily focused on samples from the southern region of the country. To comprehensively understand the genetic diversity of WBPH populations between Myanmar and Yunnan Province, China, we collected a total of 416 individual WBPH specimens from six locations in Myanmar and five locations in Yunnan Province, China. We chose the mitochondrial DNA cytochrome c oxidase subunit I (mtCOI) gene for our study because, compared to other mitochondrial genes, the mtCOI gene provides broader phylogenetic information and is considered a reliable molecular marker for evolution [23,24,25]. To gain a deeper understanding of the relationships between these individuals, we amplified the mtCOI gene from these specimens and conducted analyses on both phylogenetic relationships and population genetic structure. By delving into the genetic relationships and population structure among individual WBPH, we can gain a more comprehensive understanding of their genetic diversity and migration patterns. This study provides a scientific basis for developing more targeted cross-border monitoring and source control measures. The findings not only contribute to optimizing agricultural pest management strategies, mitigating the damage caused by WBPH to crops, but also lay a solid foundation for sustainable cross-border regional management between Myanmar and Yunnan Province, China in the future.

## 2. Materials and Methods

### 2.1. Sampling and DNA Extraction

**Figure 1:**
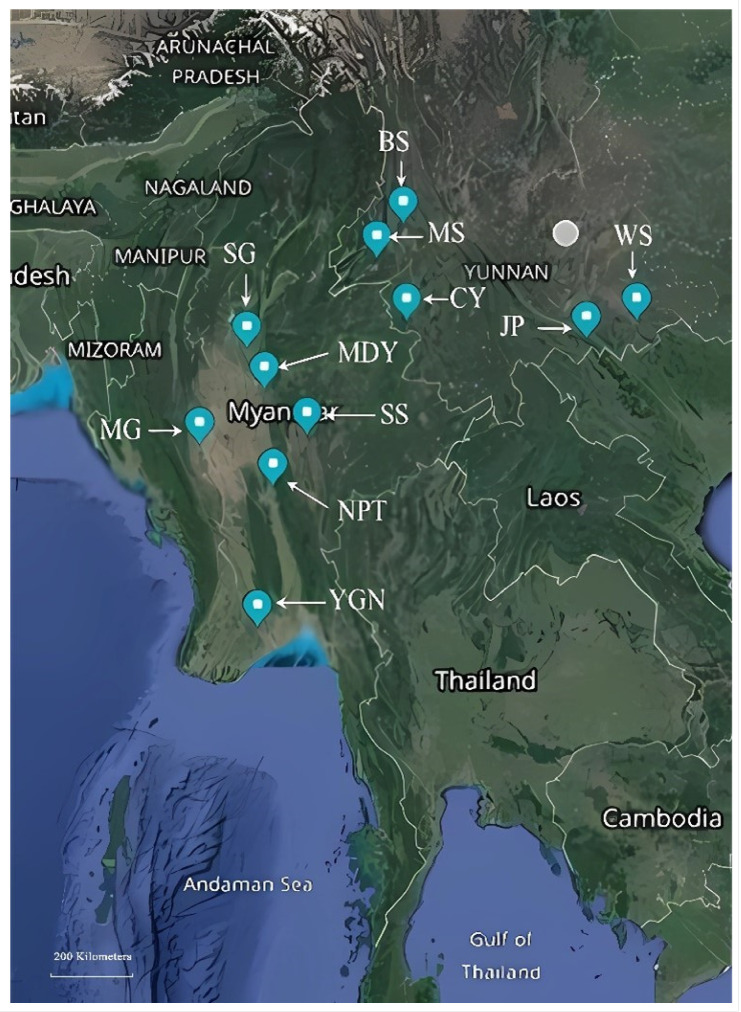
Sample collection sites of *S. furcifera* populations in Myanmar and Yunnan Province, China. The map was generated using Google Maps application (https://www.google.com/maps/, accessed on 1 November 2020).

In 2018, we collected adult *S. furcifera* samples from six locations in Myanmar and subsequently, in 2019, collected adult *S. furcifera* samples from five locations in Yunnan Province, China. The sample collection sites and population data of *S. furcifera* in Myanmar and Yunnan, China are shown in Figure 1 and Table 1. These samples were brought back to the laboratory and under controlled environmental conditions with a constant temperature of 27 ± 1 °C, relative humidity maintained at 70% ± 10%, and a light–dark cycle alternating between 14 h of light and 10 h of darkness, the WBPH population was sustained using the TN1 rice variety. During the sample preparation process, all insect samples were washed three times in 75% ethanol for 5 min each, followed by two washes in sterile ddH_2_O for 3 min each, and air-dried at room temperature [26]. Finally, genomic DNA was extracted from each individual insect using the Cwbiotech kit from Beijing, China, and the extracted DNA was stored at −20 °C.

### 2.2. Mitochondrial COI Gene Amplification and Sequencing

The *mitochondrial cytochrome oxidase subunit I* gene (mtCOI) was used to determine the genetically distinct populations of *S. furcifera.* All samples were first amplified using the mtCOI primers CI-J-2183 (5′-CAACATTTATTTTGATTTTTTGG-3′) and L2-N-3014 (5′-TCCAATGCACTAATC TGCCATATTA-3′), followed by Sanger sequencing [27]. Polymerase chain reaction (PCR) assays were performed in 20 μL of buffer containing 2 μL of 10× buffer, 1.5 mM MgCl_2_, 0.2 μM dNTPs, 1 unit of Taq DNA polymerase, 2 μL of template DNA, and 0.2 μM of each primer. PCR amplification was carried out as follows: initial denaturation at 94 °C for 5 min, followed by 35 cycles each of 30 s at 94 °C, 30 s at 55 °C, and 60 s at 72 °C, and a final elongation step at 72 °C for 10 min.

### 2.3. Population Genetic Diversity and Structure Analysis

The mtCOI sequences were aligned using Clustal W [28]. Genetic diversity indices for each population were analyzed based on mtCOI using DnaSP v. 5.0 [29]. This analysis included the number of polymorphic (segregating) sites (*S*), total number of mutations (*η*) [30], average number of nucleotide differences (*K*) [31], number of haplotypes (*H*), haplotype diversity (*Hd*), nucleotide diversity (*π*) [32], and nucleotide diversity with Jukes and Cantor correction *π* (JC) [33]. Tajima’s *D* (*D*) [34] and Fu’s *F* test [35] were performed to detect deviation from neutrality. The dispersal of different *S. furcifera* populations in Myanmar was determined by calculating the effective numbers of migrants per generation *N_e_m* using mitochondrial COI data. *N_e_m* is ϴ*M* (ϴ = *N_e_*μ, where μ is the mutation rate per site per generation; *M* = *m*/μ, where *m* is the migration rate) calculated using Bayesian search strategies in MIGRATE v. 3.2.16 software [36]. Pairwise *Fst* values and analysis of molecular variance (AMOVA) among populations were implemented with the Tamura–Nei model in Arlequin v. 3.5 [37].

Phylogenetic analyses were performed by the Bayesian inference (BI) in MrBayes v. 3.1.1 [38] and maximum likelihood (ML) methods in RAxML v. 8.2 [39]. Clustering of individuals was performed for the mtCOI dataset with BAPS v. 6.0 [40] based on the spatial clustering of groups of individual models. Twenty runs (K = 20) were made to ensure consistency and convergence of the results. The haplotype network of mtCOI genes was inferred using the median-joining algorithm [30]. In order to elucidate the relationship between haplotypes, network constructions were created using the median-joining method [41] with Network version 4.6.1.0.

### 2.4. Demographic History Analysis

To estimate the population expansion time, we utilized the Bayesian skyline plot based on the mtCOI gene in BEAST version 1.6.1 [42]. In the analysis, we used a substitution rate of 1.77% per million years [43] and the GTR + G model. The Markov Chain length was set to 300 million generations, and an uncorrelated lognormal relaxed clock model was employed, allowing rate variation among branches. During the analysis, we sampled every 1000 steps. We employed the piecewise linear skyline model with Bayesian skyline coalescent tree priors, and default values were used for other parameters. To ensure the accuracy of the results, a 10% burn-in was applied, and the results of the Bayesian skyline plot were thoroughly examined and analyzed using Tracer version 1.7 [42].

## 3. Results

### 3.1. Genetic Diversity Analysis

**Figure 2:**
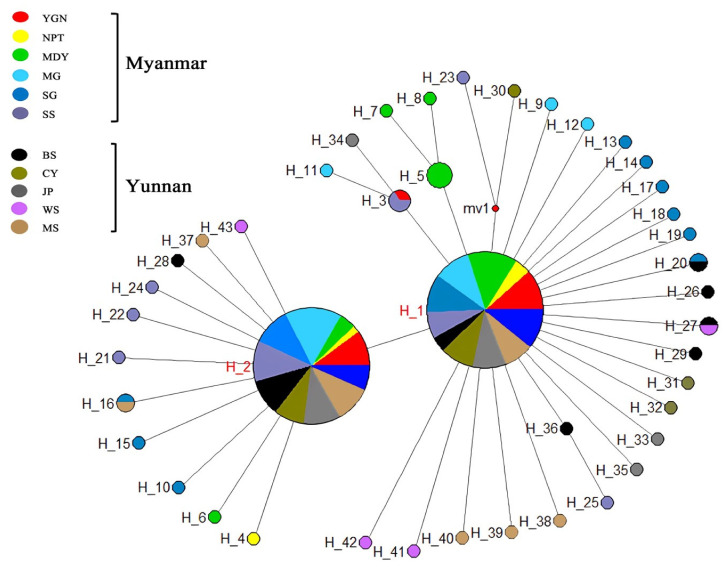
Haplotype network of the mtCOI gene. The haplotype network of COI gene sequences was inferred using the median-joining algorithm and the software Network v. 4.6.1.0. The calculated MP was used to identify and remove unnecessary median vectors and links. Each color represents a specific population.

This study identified 43 mtCOI haplotypes (designated as H_1 to H_43), as shown in Figure 2. Among these, 20 unique haplotypes were discovered in Myanmar, and 17 unique haplotypes were found in Yunnan Province, China. Additionally, six haplotypes were shared among different populations from both Myanmar and Yunnan Province as follows. (1) Two common haplotypes (H_1 and H_2) were shared by all populations in both Myanmar and Yunnan Province, China. These haplotypes were the dominant shared types in every population, with H_1 comprising approximately 62.5%, and H_2 comprising approximately 25.7% in each population. (2) H_16 was shared by the SG (Myanmar) and MS (Yunnan Province, China) populations. (3) H_20 was shared by the SG (Myanmar) and BS (Yunnan Province, China) populations. (4) The other two shared haplotypes were found in only Myanmar or Yunnan, China: H_3 was shared by the YGN (Myanmar) and SS (Myanmar) populations, while H_27 was shared by the BS (Yunnan Province, China) and WS (Yunnan Province, China) populations.

### 3.2. Population Genetic Structure and Haplotype Distribution

As shown in Table 2, we conducted an analysis of genetic diversity among 11 different populations based on the mtCOI gene. The results revealed that in Yunnan Province, China, and northern Myanmar, the number of haplotypes (*H*) was more abundant compared to the central part of Myanmar (NPT) and the southern part of Myanmar (YGN). The haplotype diversity (*Hd*) among populations in Myanmar ranged from 0.362 (NPT) to 0.662 (SS), while in Yunnan Province, China, it ranged from 0.488 (WS) to 0.662 (BS). The average *Hd* value in Myanmar (0.589) was not significantly different from that in Yunnan Province, China (0.571). Nine of the eleven populations, except for those from the Yangon and Naypyitaw regions in Myanmar, had negative Fu’s *F* and Tajima’s *D* indices (*p* < 0.05). These results indicated a recent population expansion following a bottleneck event.

Nine of fifty-five pairwise *Fst* values based on the mitochondrial gene were significant (*p* < 0.05), such as the differentiation between YGN and BS (*Fst* = 0.02709, *p* < 0.05); SS and WS (*Fst* = 0.06724, *p* < 0.05); and MDY and YGN, MG, SG, SS, BS, JP, and MS (*Fst* = 0.03923, 0.00080, 0.02784, 0.00056, 0.00051, 0.01922, 0.00908, respectively, *p* < 0.05). The highest *Fst* value (*Fst* = 0.98360, *p* > 0.05) was observed between the YGN population and SG population. Most pairwise *Fst* values were not significant (*p* > 0.05), which suggested high-level gene flow among the populations in Myanmar and Yunnan Province, China (Table 3).

**Figure 3:**
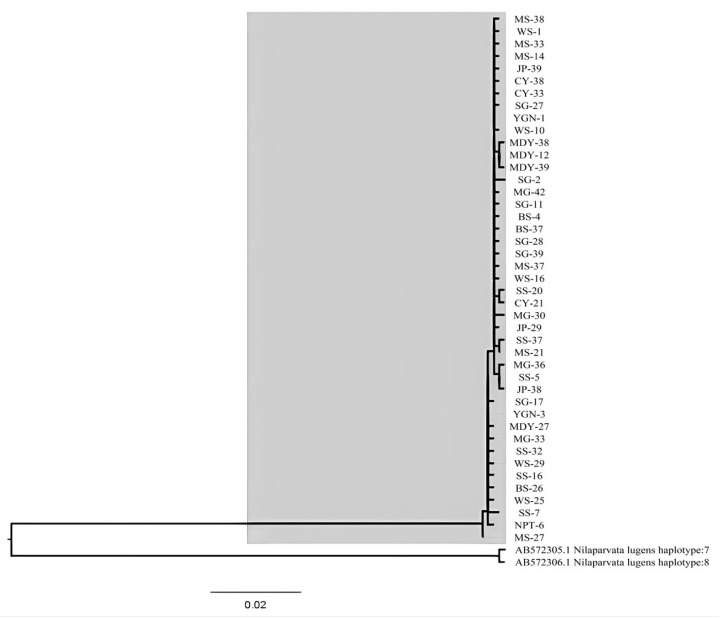
Maximum likelihood tree based on mitochondrial haplotypes of *S. furcifera.* The gray background represents the white-backed planthopper, while the other two sequences represent the brown planthopper.

The analysis of molecular variance (AMOVA) revealed that 98.7% of the variation was attributed to differences within populations (*p* < 0.05), 1.9% of the variation was due to differences among populations within the same region (*p* < 0.05), and −0.55% of the variation was attributed to differences among different regions (*p* > 0.05). On the contrary, ΦCT was not significant, indicating no difference in the genetic structure among the *S. furcifera* populations in Myanmar and Yunnan Province of China (Table 4). The maximum likelihood (ML) tree topology showed that there were no significant genealogical branches for all *S. furcifera* populations in Myanmar and Yunnan Province (Figure 3).

### 3.3. Gene Flow Based on Mitochondrial DNA

**Figure 4:**
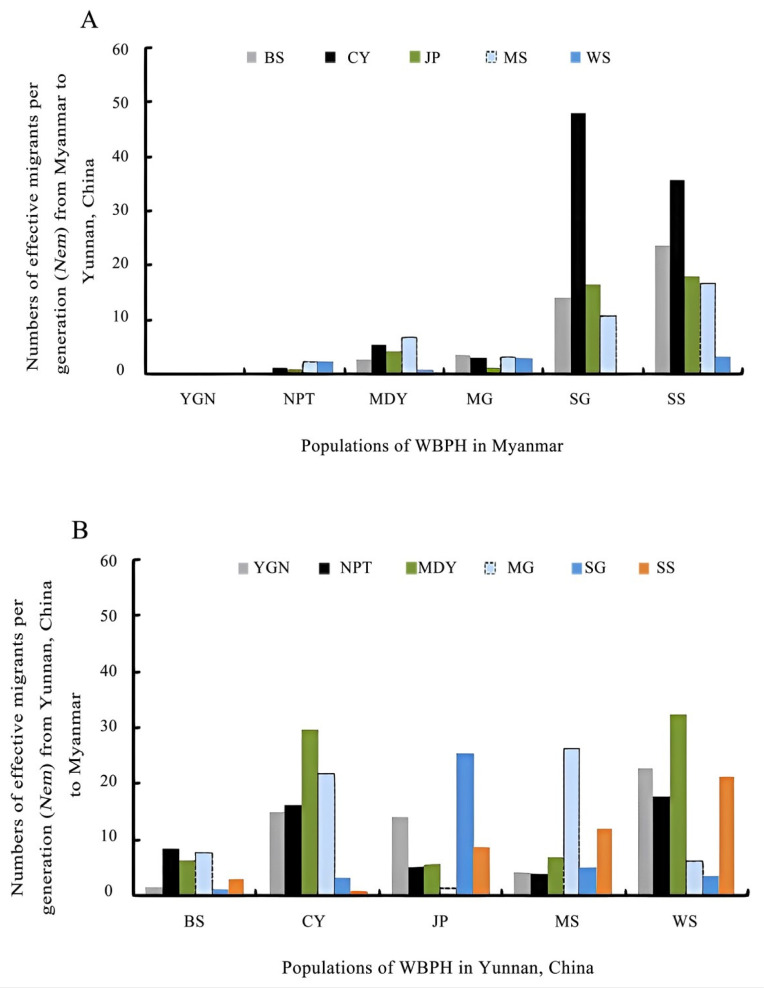
Comparison of the effective migrants per generation (*N_e_m*) of *S. furcifera* populations in Myanmar (**A**) and Yunnan Province, China (**B**). The eleven populations were grouped into six populations in Myanmar and five populations in Yunnan Province.

The unidirectional estimates of total migrants (*N_e_m*) ranged from 0.0254 (from YGN to SG, and YGN to BS) to 51.3476 (from SS to JP). The highest numbers of total migrants (*N_e_m* > 200) were found in some populations of Myanmar (SS 255.15, MG 235.52, and SG 202.46), while moderate numbers of total migrants (*N_e_m* > 100) were found in some populations of Yunnan Province, China (CY 178.71, WS 169.78, MS 129.30, and JP 108.04). The lowest numbers of total migrants were found in Mandalay Division, Myanmar (MDY 21.80) and Baoshan, Yunnan Province, China (BS 50.29). The total number of migrants (*N_e_m*) was less in southern Myanmar, e.g., from YGN to CY (0.035), JP (0.029), MS (0.034), and WS (0.050) (Yunnan Province, China) compared with that in north and northeast Myanmar, which was from MG to CY (47.96), from SG to CY (35.77), from SS to JP (51.35), and from SS to WS (37.11), indicating that intensive gene flow existed among north and northeast Myanmar and Yunnan Province, China (Figure 4, Table 5).

In Myanmar, where six populations (YGN, NPT, MDY, MG, SG, SS) were identified, and in Yunnan Province, China, where five populations (BS, CY, JP, MS, WS) were studied, it was observed that the effective migration values in the northern and northeastern regions of Myanmar (SG, SS) were higher than those in the central and southern parts of Myanmar (YGN, NPT). Simultaneously, in the southwestern (BS, CY) and southern (JP) regions of Yunnan Province, the effective immigrant values (immigration from Myanmar) were higher than the effective emigrant values (emigration to Myanmar) (Figure 4). By comparing the migration values in Myanmar and Yunnan Province, China, it can be inferred that north and northeast Myanmar were the main migration sources of WBPH populations in southwest and south Yunnan Province.

### 3.4. Demographic History Analysis

**Figure 5:**
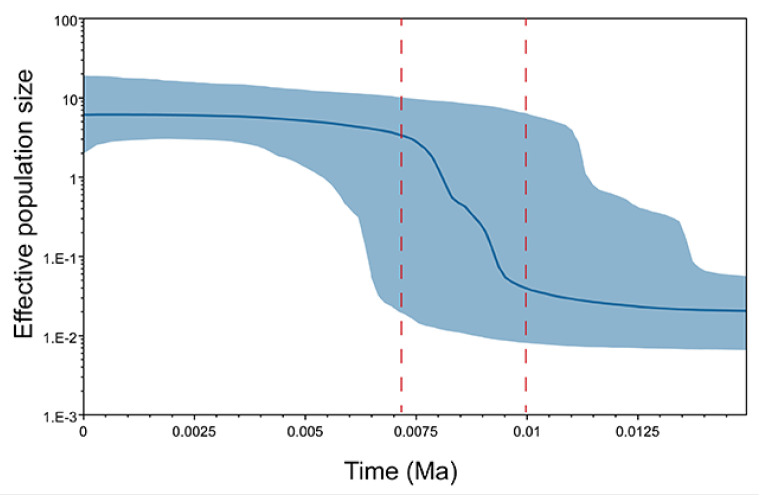
The demographic history of *S. furcifera* was reconstructed through Bayesian skyline plots utilizing the mtCOI gene with a substitution rate of 0.0177. The *X*-axis represents the timescale in the past, while the *Y*-axis displays the estimated effective population size. Solid curves indicate the median effective population size, and the shaded range represents the 95% highest posterior density (HPD) intervals.

Five out of the eleven populations showed negative Fu’s *F* indices with statistical significance (*p* < 0.05), indicating recent demographic expansion in *S. furcifera* (Table 2). These findings were consistent with the results obtained from the Bayesian skyline plot (BSP) analysis. The BSP analysis, conducted with a substitution rate of 1.77% per million years based on the mtCOI gene, revealed that the effective population size of the WBPH expanded approximately 1000 to 7000 years ago (Figure 5).

## 4. Discussion

Mitochondrial genes are extremely suitable for tracing the history of populations, estimation of migration, and gene flow [44,45]. Since the mitochondria of most insects are haploid and inherited maternally, the mitochondrial genome is transmitted clonally [46]. In contrast, the nuclear genome is diploid with biparental inheritance. Therefore, mitochondrial DNA variations allow for more precise tracking of recent population histories compared to nuclear loci [47]. In this study, 417 individuals were sampled from six Provinces in Myanmar and five sites in Yunnan Province, China. The mtCOI was used to estimate genetic variations among geographic populations of *S. furcifera* in Myanmar and Yunnan Province, China. Among the confirmed 43 mtCOI haplotypes, haplotypes H_1 and H_2 were found to be widely distributed in all populations of *S. furcifera* in Myanmar and Yunnan Province, China. This suggests extensive gene flow among these populations. In addition, H_16 was shared by the SG (Myanmar) and MS (Yunnan Province, China) populations and H_20 was shared by the SG (Myanmar) and BS (Yunnan Province, China) populations, indicating a close relationship between WBPH populations from north Myanmar and southwest–south Yunnan Province, China. Matsumoto et al. identified 20 WBPH haplotypes based on the COI fragment [19], 23 haplotypes less than present study, possibly due to fewer samples being tested (20 individuals per population) and lacking samples collected from Myanmar and Yunnan Province.

Genetic variations were found in migratory insects; the population’s genetic structure can be affected by evolutionary factors, such as mating system, gene flow, mode of reproduction, and natural selection [48]. Previous studies demonstrated that Myanmar was one of the possible migration sources of *S. furcifera* to Yunnan Province, China [13,20]. In the present study, the total number of migrants (*N_e_m*) from the YGN, NPT, MDY, MG, SG, and SS populations to Yunnan Province, China, was 7.4, 18.0, 13.1, 79.0, 88.1, and 133.2, respectively. Furthermore, the SG of Myanmar and the CY of Yunnan Province, China had the highest *N_e_m* value, while the SS of Myanmar and the JP of Yunnan Province, China had the highest *N_e_m* value. All these results suggested that north Myanmar (Sagaing Division and Shan State) might be the major immigration source of the populations in southwestern (e.g., MS and CY) and southern (JP) Yunnan Province, China. The results also confirmed the previous findings on the migration simulations of *S. furcifera* in the Greater Mekong Subregion (GMS) countries [7].

Temperature stress has adverse effects on the survival and reproduction of the WBPH, explaining their adoption of long-distance migration strategies [49]. Under low-temperature conditions (5 °C) after 48 h of treatment, the mortality rate significantly increased (>70%), much higher than the mortality rate under optimal temperature conditions (25 °C) (<5%) [50]. This indicates that the WBPH lacks cold tolerance. Additionally, wind is considered a vector for many migrating organisms, playing a crucial role in their migration process [51]. Every spring, driven by the southwest monsoon, the WBPH migrates northward and can even reach northern China, Japan, and Korea [52]. Analysis based on the mtCOI dataset suggests that the *S. furcifera* population began to expand around 10,000 years ago, which aligns with the results of population expansion after the last glacial period. With the warming climate after the last glacial period, the number of suitable habitats gradually increased. Our study findings are consistent with research conclusions regarding classical expansion patterns after the last glacial period. Similar patterns have been observed in other insects, such as *Stomoxys calcitrans* [53], *Locusta migratoria* [54], *Megacopta cribraria* [55], and *Nesidiocoris tenuis* [56].

Understanding the migration patterns of significant agricultural pests is crucial for developing sustainable pest management strategies. Failure to promptly implement scientifically effective control measures can result in incalculable damage to crops during large-scale migrations, leading to severe economic problems [57,58,59]. Moreover, such massive pest migrations can trigger serious ecological issues. During their migration, pests may attack various plants, not just limited to crops, but may also pose a threat to wild vegetation, disrupting the balance of natural ecosystems. Therefore, understanding the migration pattern of pests and adopting scientific and reasonable pest management strategies are of vital significance for maintaining ecological balance, guaranteeing the safe production of crops and realizing the sustainable development of agriculture.

## 5. Conclusions

This study revealed that north and northeast Myanmar might be the major WBPH immigration source of the populations in southwestern and southern Yunnan Province, China. These findings might be useful in designing regional control programs for WBPH populations in these regions.

## Figures and Tables

**Table 1:** Sampling information of *S. furcifera* collected from various locations in Myanmar and Yunnan Province, China.

Code	Location	Longitude	Latitude	Date
YGN	Tontay, Yangon Division, Myanmar	16.71	95.94	5 September 2018
NPT	Pyinmana, Naypyitaw, Myanmar	19.71	96.28	10 May 2018
MG	Salin, Magway Division, Myanmar	20.58	94.65	8 June 2018
SG	Shwebo, Sagaing Division, Myanmar	22.58	95.70	15 May 2018
MDY	Sintkaing, Mandalay Division, Myanmar	21.74	96.09	11 April 2018
SS	Taunggyi, Shan State, Myanmar	20.91	96.94	10 June 2019
BS	Baoshan, Yunnan, China	25.13	99.17	10 May 2019
CY	Cangyuan, Yunnan, China	23.15	99.25	17 April 2019
JP	Jinping, Yunnan, China	22.78	103.23	15 May 2019
MS	Mangshi, Yunnan, China	24.44	98.58	10 May 2019
WS	Wenshan, Yunnan, China	23.16	104.35	5 May 2019

**Table 2:** Genetic diversity indices of *S. furcifera* populations in Myanmar and Yunnan Province, China.

Population Code (No. of Individuals Tested)	*S*	*η*	*H*	*D* (*p*)	*Fs* (*p*)	*Hd* (SD)	*π* (SD)	*K*	*π* (JC)
YGN (43)	2	2	3	−0.10389 (*p* > 0.10)	0.011 (0.280)	0.424 (0.068)	0.00055 (0.00010)	0.436	0.00055
NPT(15)	2	2	3	−0.59419 (*p* > 0.10)	0.518 (0.251)	0.362 (0.145)	0.00060 (0.00027)	0.476	0.00060
MDY (48)	5	5	6	−1.20378 (*p* > 0.10)	−2.582 (0.039)	0.427 (0.085)	0.00072 (0.00017)	0.572	0.00073
MG (47)	6	6	6	−1.24044 (*p* > 0.10)	−2.187 (0.067)	0.574 (0.047)	0.00089 (0.00014)	0.705	0.00090
SG (47)	10	10	10	−1.88055 (*p* < 0.05)	−7.005 (0.001)	0.599 (0.068)	0.00100 (0.00020)	0.792	0.00100
SS (38)	10	10	8	−1.72907 (*p* > 0.10)	−3.424 (0.022)	0.662 (0.060)	0.00104 (0.00017)	0.825	0.00129
BS (26)	5	5	6	−1.05038 (*p* > 0.10)	−2.447 (0.056)	0.662 (0.060)	0.00104 (0.00017)	0.825	0.00105
CY (36)	5	5	5	−1.29300 (*p* > 0.05)	−1.767 (0.098)	0.505 (0.078)	0.00077 (0.00017)	0.608	0.00077
JP (39)	5	5	5	−1.21593 (*p* > 0.10)	−1.632 (0.107)	0.521 (0.068)	0.00079 (0.00015)	0.621	0.00079
MS (38)	7	7	8	−1.52318 (*p* > 0.05)	−4.725 (0.007)	0.623 (0.065)	0.00096 (0.00015)	0.760	0.00096
WS (40)	6	6	7	−1.51888 (*p* > 0.10)	−4.204 (0.011)	0.488 (0.087)	0.00077 (0.00017)	0.608	0.00077

*S*, number of polymorphic (segregating) sites; *η*, total number of mutations; *H*, number of haplotypes; *Hd*, haplotype diversity; ***π***, nucleotide diversity; *K*, average number of nucleotide differences; *π* (JC), nucleotide diversity with Jukes and Cantor correction; *D*, Tajima’s *D* statistic; *Fs*, Fu’s *F* test statistic; *p*, significance values of the parameters evaluated using 10,000 simulations.

**Table 3:** Pairwise *Fst* values (*p* value) for *S. furcifera* populations in Myanmar and Yunnan Province, China.

	YGN	NPT	MDY	MG	SG	SS	BS	CY	JP	MS	WS
YGN	---										
NPT	0.33603	---									
MDY	0.03923 *	0.62196	---								
MG	0.27267	0.28552	0.00080 *	---							
SG	0.98360	0.83533	0.02784 *	0.25266	---						
SS	0.29515	0.44171	0.00056 *	0.23759	0.30503	---					
BS	0.02709 *	0.09100	0.00051 *	0.25605	0.23489	0.50366	---				
CY	0.55549	0.56302	0.06487	0.41749	0.98038	0.27200	0.06905	---			
JP	0.60843	0.45765	0.01922 *	0.54649	0.99198	0.30037	0.12561	0.85094	---		
MS	0.31108	0.73535	0.00908 *	0.43340	0.87622	0.58144	0.39849	0.68066	0.64490	---	
WS	0.38822	0.88951	0.11603	0.08123	0.83152	0.06724 *	0.04464	0.68255	0.38785	0.26380	---

* Significant values at 0.05 level.

**Table 4:** AMOVA analysis of *S. furcifera* populations in Myanmar and Yunnan Province, China.

Source of Variation	Sum of Squares	Variance Components	Percentage Variation (*p* Value)
Among areas (ΦCT)	0.203	−0.00189	−0.55% (0.90029)
Among population within areas (ΦSC)	5.208	0.00638	1.85% (0.02639)
Within population (ΦST)	137.766	0.33933	98.69% (0.03519)

**Table 5:** Numbers of effective migrants per generation (*N_e_m*) in the *S. furcifera* populations found in Myanmar and Yunnan Province, China.

Population Code (*ἰ*)	*ϴἰ*	YGN→*ἰ*	NPT→*ἰ*	MDY→*ἰ*	MG→*ἰ*	SG→*ἰ*	SS→*ἰ*	BS→*ἰ*	CY→*ἰ*	JP→*ἰ*	MS→*ἰ*	WS→*ἰ*	∑(immigration)
YGN	0.0769	---	4.8837	2.0503	50.8836	35.6291	49.9904	1.4941	14.9216	14.2943	4.3621	22.7011	201.2103
NPT	0.0070	19.7556	---	3.1556	0.0284	25.2077	5.8986	8.5400	16.3450	5.3087	4.0499	17.8145	106.104
MDY	0.0033	2.0240	1.7057	---	8.6284	35.7985	13.5892	6.5054	29.7385	5.7505	7.1291	32.4422	143.3115
MG	0.0860	4.0226	4.2957	1.9391	---	6.5406	23.6466	7.6845	21.8516	1.5264	26.3409	6.1254	103.9734
SG	0.0363	0.0254	0.3617	1.4617	73.4156	---	28.8666	1.1993	3.1616	25.4497	5.1209	3.6489	142.7114
SS	0.0522	2.9464	2.3777	0.0534	23.5356	11.2074	---	3.2281	1.0616	8.8223	12.0602	21.2152	86.5079
BS	0.0087	0.0254	0.4643	2.6978	3.8124	14.1333	23.7510	---	22.1785	12.7008	25.2923	7.5781	112.6339
CY	0.0350	1.4090	5.5603	3.1468	47.9596	35.7742	2.6972	8.2047	---	5.8271	7.1291	19.4985	137.2065
JP	0.0288	0.8456	4.0950	1.1783	16.4836	18.1713	51.3476	1.1011	33.6350	---	13.2876	19.9606	160.1057
MS	0.0335	2.5882	6.8810	3.2079	10.7500	16.7448	18.2528	8.5573	33.0050	0.0095	---	18.8051	118.8016
WS	0.0495	2.4852	0.9777	2.9136	0.0284	3.2523	37.1142	3.7773	2.8116	28.3487	24.5335	---	106.2425
∑(emigration)	---	36.1274	31.6028	21.8045	235.5256	202.4592	255.1542	50.2918	178.7100	108.0380	129.3056	169.7896	---

## Data Availability

Data are contained within the article.
